# Emotions and perceptions of elderly patients during the care pathway in healthcare settings

**DOI:** 10.1192/j.eurpsy.2025.1730

**Published:** 2025-08-26

**Authors:** F. Franza, A. Franza, G. Conte, L. Roselli, B. Solomita, M. Minò, A. Vacca

**Affiliations:** 1 ”Villa dei Pini”, Psychiatric rehabilitation center; 2 Neamente Neuroscience Association, Avellino; 3 Il Filo di Arianna” –Alzheimer’s Disease Center Social Cooperative, Venosa; 4 Healthcare Residence, RASS1 “Villa Caterina”, Pescopagano (PZ); 5 Psychiatric Rehabilitation Center “Don Tonino Bello” - Assoc. M.I.T.A.G. - Onlus, Brindisi; 6 Mental Health Department, ASL Taranto, Grottaglie - Manduria, Italy

## Abstract

**Introduction:**

The number of elderly people requiring social and health care support in the last years of life is constantly increasing. The consequence is a significant increase in the number of guests in assisted nursing homes. The elderly patient, collaborating with healthcare professionals, becomes the protagonist of the entire care process. The places and times of care, together with the lived experience and the emotions felt, are just some dimensions of humanization, dignity of care and dignified care. This recognition of the patient experience within the definition of quality of care is associated with better clinical outcomes and patient safety. Patient Reported Experience Measures (PREM) are psychometrically validated questionnaires returned directly by patients and aim to provide a standardized assessment of individual care experiences.

**Objectives:**

Assess the emotionality, dignity, depressive symptoms of the elderly patient without significant psychiatric disorders at the time of admission to a place of care.

**Methods:**

Sixty-seven elderly subjects (28 F, 38 M) were recruited in some elderly residential facilities (total mean age (yrs)(*±SD*): 75.64 *±5.96*)

**Inclusion criteria:**

age ≥ 65 years; MMSE ≥20; absence of overt diagnosis of psychiatric or neurodegenerative disorders (evaluation with SCID-5-CV). All patients were given a PREM questionnaire at the beginning and during the care pathway. All patients were administered at baseline (T0), after 6 months (T1) and after 1 year (T2) the following evaluation rating scales:

- Mini-Mental State Examination (MMSE) (only T0); Patient Dignity Inventory (PDI); Geriatric Depression Scale (GDS); Global Assessment of Functioning (GAF); Quality Life Index (QLi)

The data were statistically analyzed with the EZAnalyze 3.0 software for the Excel platform.

**Results:**

Tables 1 and 2 show the results obtained with each scale analyzed. On the PDI scale, the ANOVA results indicate that at least two of the repeated measures differed significantly [Mean scores *± Std. Dev*: (T0) 63.388 *± 22.042*; (T1): 57.313 *±21.159*; (T2): 49.985 *±17.418*]. The data obtained with the GDS scale showed no variation during the observational period. Although the differences were not statistically significant, the data indicate that no increases in depressive symptoms were observed. I results obtained with the QLi showed that the ANOVA results indicate that at least two of the repeated measures differed significantly [Mean scores *± SD:* (T0) 3.358 *± 1.164*; (T1): 6.075 *±1.222*; (T2): 6.657 *±1.213].* Similar results were observed with the GAF scale.

**Image 1:**

**Figure:**
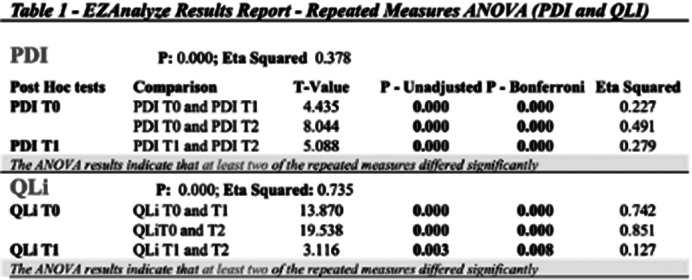

**Image 2:**

**Figure:**
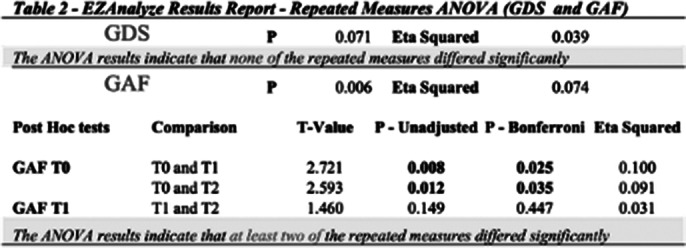

**Conclusions:**

Intervention programs that allow older residents to express their emotions and observations are not only beneficial for corporate welfare, but also promote a sense of empowerment and involvement. Our small observational study has shown that these programs can significantly improve residents’ quality of life and protect against the onset of depressive symptoms.

**Disclosure of Interest:**

None Declared

